# 

**Published:** 1918-06-22

**Authors:** 


### CONTENTS.

PAGE

**The Churches and the War: Hospital Sunday** 241

**The Claims of Qualified Dentistry** ... ... 242

**Hospital and Institutional News** ... ... ... 243

Increase in Price—Special Notice—American Wounded at Portsmouth—A Canadian Hospital Founder—The Danger of Unqualified Persons on Hospital Staffs—Lord Knutsford's Sermon—Tombolas and Hospital Ethics—American Assistance at York—A Convalescent Home for Discharged Men—American Navy's Hospital in London—A Red Cross Restaurant—The Salaries of Women Surgeons—A Hospital Explosion at Birmingham—"Sacrilege" Averted at a Military Hospital—The Spanish Epidemic—A Professorship Without a Title—M. de Leval and Nurse Cavell—Presentation to a Woman Hospital Secretary—A Woman Medical Officer of Health—Death of a Prominent Public Pharmacist—Farm Colonies for Consumptives—A Proposed Asylum's Conference—Birmingham's New Infant Hospital—This Week's Drug Market

**The Great War** ... ... ... ... ... ... 247

IX. Flies and Disease.

**A Consultation of Physicians** ... ... ... 248

Designed by Hogarth.

**Dr. Mercier and Asylum Workers** ... ... 249

A Mansion House Speech and Some Reminiscences.

**Hospitals in Holy Land** ... ... ... ... 251

Some Foreign Hospitals in Jerusalem.

PAGE

**The Economics of Nursing** ... ... ... ... 253

Lord Knutsford's Reply to "Ierne's" Shylock's Article.

**Nursing Affairs in Ireland** ... ... ... ... 254

The Hon. Sir Arthur Stanley's Visit.

**The Matrons' and Sisters' Department** ... 255

District Nursing: II. Some Aspects of the Work.

Round the Hospitals.

Nurses' Tribute Fund in Ireland—Its Enthusiastic Welcome—Nurses Who Died for Their Country (Illustrated)—Trained Nurses and Housekeeping—Important New Departure, Middlesex Hospital—School Nurses' Work and Training.

**Royal British Nurses' Association** ... ... 259

The Annual Meeting.

**Matrons' and Sisters' Appointments** ... .. 259

**Parliament and the Profession** ... ... 260

**Changes at the Pharmaceutical Society** ... 260

**How to Revive Church Life** ... ... ... 261

The Annual Reminder of Hospital Sunday.

**The Voluntary Hospitals' Budget** ... ... 262

**Over 1,700,000 Sufferers Helped by our Hospitals** ... ... ... ... ... ... 263

A Single Year's Roll-call of the Sick.

**Hospitals and their Special Needs** ... ... 265

**The Institutional Worker** ... ... (Suppl.) 1

